# Spinal infections

**DOI:** 10.3389/fnins.2026.1864932

**Published:** 2026-06-24

**Authors:** Huozong Cao, Feng Xue

**Affiliations:** Peking University People's Hospital, Beijing, China

**Keywords:** diagnosis, pyogenic spondylitis, spinal infection, spondylodiscitis, treatment, tuberculous spondylitis

## Abstract

Spinal infection is a serious and increasingly concerning condition within orthopedics and infectious diseases, with a gradually rising incidence in recent years. This trend is closely associated with the aging population, an increase in invasive spinal diagnostic and therapeutic procedures, a growing immunocompromised population, and the prevalence of drug-resistant strains. Due to the atypical early symptoms of spinal infection, along with the diversity of causative pathogens and the deep-seated location of the infection, clinical misdiagnosis or delayed treatment is common. In severe cases, spinal infection can lead to vertebral destruction, spinal deformity, neurological impairment, and even paralysis, imposing a heavy burden on patients and society. This article aims to systematically summarize the current general understanding of spinal infection, with an emphasis on its epidemiological characteristics, distribution of common pathogens, typical and atypical clinical manifestations, the diagnostic value of imaging and laboratory tests, strategies for etiological identification, and the principles of selecting medical versus surgical treatment. By integrating existing evidence and clinical experience, this review is expected to provide clinicians with a systematic diagnostic approach and treatment reference when facing suspected or confirmed cases of spinal infection, thereby improving early recognition and patient prognosis.

## 1 Introduction

Spinal infection is a complex and severe disease caused by bacteria, fungi and parasites, which can lead to lesions in the vertebral bodies, intervertebral discs, soft tissues and other structures (Duarte and Vaccaro, 2013; Babic and Simpfendorfer, 2017; Lener et al., 2018). The spinal cord can also be involved in some cases, and spinal cord injury is permanent, which will cause great damage to patients’ daily life and serve as a major cause of long-term disability and death. However, recent studies have shown that functional recovery of patients can be promoted through 3D printing technology. But it is not within the main scope of this paper (Bhattacharyya and Bradshaw, 2021; Wang et al., 2021; Wang et al., 2025). In the past, spinal infection was regarded as a rare disease, but in recent years, its incidence has gradually increased. This may be related to the aging population, the increasing number of diabetic patients, the abuse of intravenous drugs, the growing number of patients with immunodeficiency diseases such as AIDS, and the continuous improvement of detection and diagnostic levels (Jensen et al., 1997; Rigamonti et al., 1999; Chuo et al., 2007; Chakaya et al., 2021). Germany recorded patients with vertebral osteomyelitis from 2005 to 2019, and the incidence rose from 5.5 per 100,000 in 2005 to 12.4 per 100,000 in 2019. In France, the incidence increased from 6.1 per 100,000 in 2010 to 11.3 per 100,000 in 2019. Relevant studies have shown that the incidence of vertebral osteomyelitis in the United States increased from 2.9 per 100,000 in 1998 to 5.4 per 100,000 in 2013. The incidence in Asian countries is also on the rise. For instance, the figure in Japan climbed from 5.3 per 100,000 in 2007 to 7.4 per 100,000 in 2010 (Akiyama et al., 2013; Issa et al., 2018; Conan et al., 2021; Heck et al., 2024). The age distribution of patients with spinal infection presents a bimodal pattern, with the most prevalent onset ranges being 10–20 years old and 50–70 years old. The disease also shows age susceptibility. Current studies have demonstrated a higher incidence in males, with a male-to-female prevalence ratio of approximately 2–5:1 (Jevtic, 2004; Duarte and Vaccaro, 2013; Lener et al., 2018; Baryeh et al., 2022; Laur et al., 2024). In the past, *Mycobacterium tuberculosis* was the most common cause of spinal infection, while *Staphylococcus aureus* has gradually become the predominant pathogenic bacterium at present (Calderone and Larsen, 1996; Stangenberg et al., 2021). Geographical factors also affect the incidence of different pathogenic bacteria. *Mycobacterium tuberculosis* remains prevalent in developing countries with a far higher incidence than that in developed countries, whereas *Staphylococcus aureus* is the most common pathogen in developed countries (Calderone and Larsen, 1996; Bhattacharyya and Bradshaw, 2021; Shanmuganathan et al., 2023; Yu et al., 2025). Spinal infections can be classified into typical and atypical spinal infections based on different pathogens. Generally, those caused by pyogenic bacteria are defined as typical spinal infections, while those caused by other pathogens are classified as atypical spinal infections. The most common clinical manifestation of both is back pain, and the thoracolumbar and lumbar regions are the most frequently involved sites. However, atypical spinal infections may present some specific clinical manifestations due to the particularity of pathogens. For example, vertebral osteomyelitis associated with cat-scratch disease may manifest as atlantoaxial instability, torticollis or fever (Gonzalez et al., 2023; Yu et al., 2025; Zou et al., 2025).

## 2 Classification

### 2.1 Typical spinal infection: typical spinal infection is caused by pyogenic bacteria

The most common pathogen is usually *Staphylococcus aureus*, accounting for more than 50% of cases, followed by Enterococcus and Streptococcus. Infections caused by anaerobic bacteria are rare, accounting for less than 5% (Legrand et al., 2001; Cebrián Parra et al., 2012; Loibl et al., 2014). However, it has been reported that the infection rate of Salmonella is relatively high in patients with sickle cell disease, and the infection rate of *Pseudomonas aeruginosa* is higher in patients with intravenous drug abuse (Go et al., 2012). The onset age is usually relatively advanced, with a predominance in males, and the male-to-female ratio is approximately 1.5–2:1. The most common route of infection is hematogenous spread, while direct inoculation and extension from adjacent tissues are less common (Pola et al., 2017). Unlike mycobacterial infections that typically follow a chronic course, infections caused by pyogenic bacteria may be acute (Bhattacharyya and Bradshaw, 2021). The lumbar spine is the most commonly involved site, accounting for approximately 50–60% of cases, followed by the thoracic spine (25–33%), and the cervical spine is the least frequently affected (less than 10–15%). Moreover, pyogenic infections mostly involve a single spinal segment (Henry et al., 2025). The most common symptom is back pain, which occurs in more than 80% of patients. The pain is usually localized to the infected site but may also radiate to the extremities, groin, and other regions (Zimmerli, 2010; Saeed et al., 2019; Tsantes et al., 2020). The second most common symptom is fever, which does not occur in most patients. Reports indicate that only about 35–60% of patients present with fever. Therefore, the absence of fever does not rule out the diagnosis of pyogenic spondylodiscitis (Zimmerli, 2010). In addition, patients may present with systemic symptoms such as anorexia, weight loss, and fatigue. However, these symptoms are nonspecific and offer little help for diagnosis (Cornett et al., 2016).

### 2.2 Atypical spinal infection

#### 2.2.1 Tuberculous spondylitis

*Mycobacterium tuberculosis* usually affects the lungs, but can also cause extrapulmonary manifestations. Bone tuberculosis accounts for 10% of extrapulmonary tuberculosis, among which spinal tuberculosis accounts for 50% (Rajasekaran et al., 2018). Globally, 11 million people are infected with tuberculosis, and approximately 150,000 new cases of spinal tuberculosis occur each year. Spinal tuberculosis is more common in developing countries. Reported cases from Pakistan, China, Nigeria, India, Indonesia and South Africa account for 64% of the global total. The risk of tuberculosis infection in HIV-infected patients is significantly higher than in healthy individuals, with a reported 21–30-fold increased risk. Unlike pyogenic infections, *Mycobacterium tuberculosis* infects the spine only through hematogenous spread. Systemic symptoms such as fever and loss of appetite are usually the initial manifestations of spinal tuberculosis. Currently, it still takes approximately 3–6 months from symptom onset to confirmed diagnosis. Back pain is the most common symptom, occurring in 90–100% of patients several weeks before presentation, and is usually localized to the affected area. Neurological deficits, progressive vertebral collapse and spinal deformity are typical features of spinal tuberculosis (Shanmuganathan et al., 2023). A retrospective study including 1,652 patients showed that spinal tuberculosis most frequently involved the lumbar spine (37%), with the peripheral type being the most common (82%). The most prevalent age group was 21–30 years (33%), and there was almost no difference in prevalence between males and females (1.13:1). Thirty-two percent of patients had one or more comorbidities, the most common being hypertension (11.8%) and diabetes mellitus (9.2%), while only 1.1% were HIV-positive, which may be related to the avoidance of mainstream medical care among such patients. Axial pain was the most common symptom (98%), with a mean delay of 4.5 months from symptom onset to diagnosis (Garg et al., 2022).

#### 2.2.2 Nontuberculous mycobacterial spondylitis

Nontuberculous mycobacteria refer to mycobacteria other than *Mycobacterium tuberculosis* and *Mycobacterium leprae*. More than 190 species of nontuberculous mycobacteria have been identified to date. Some are non-pathogenic, while others can cause difficult-to-treat infections once established. *Mycobacterium avium* is a well-known species of nontuberculous mycobacteria (Tortoli, 2014; Tan and Kasperbauer, 2021). The lungs are the most commonly infected site, but other sites such as the skin, bones, and lymph nodes may also be affected (Tortoli, 2009). Nontuberculous mycobacteria have a unique mode of transmission. Unlike *Mycobacterium tuberculosis* and other bacteria, they are not transmitted from person to person. Humans can only become infected by exposure to nontuberculous mycobacteria in the environment, which may be one reason for their relatively low incidence (Feazel et al., 2009; Falkinham, 2011; Wallace et al., 2013). Nontuberculous mycobacteria are widespread in the human living environment, with water and soil being common environmental sources (Falkinham, 2015). Nontuberculous mycobacteria can survive in harsh conditions, such as exposure to antibiotics and disinfectants, high temperatures, and the low pH environment of the stomach. This may be due to their thin peptidoglycan layer surrounded by a thick lipid-rich outer membrane (Portaels and Pattyn, 1982; Schulze-Röbbecke and Buchholtz, 1992; Brennan and Nikaido, 1995; Bodmer et al., 2000; Falkinham, 2009). Nontuberculous mycobacteria are also usually transmitted hematogenously and require macrophages as intermediaries (Petitjean et al., 2004). A study on the prevalence of nontuberculous mycobacterial infections was conducted in North America in 2012. It showed that the overall prevalence was approximately 112 per 100,000 population, with a markedly higher prevalence in females than in males. This is consistent with the gender ratio reported in New Zealand, where infections are also more common in women (Tan and Kasperbauer, 2021). Statistics indicate that the global prevalence of *Mycobacterium avium* infection increased by 0.5 to 2.6 times between 2007 and 2014. Most affected patients were immunocompromised individuals, with associated conditions including diabetes mellitus, liver disease, cardiovascular disease, connective tissue disease, and corticosteroid use. Studies suggest that corticosteroid use is the most common predisposing factor. The clinical manifestations of nontuberculous mycobacterial infection are atypical, such as back pain or systemic symptoms. Due to its slow and insidious progression, the disease is often misdiagnosed as a malignant tumor or missed entirely (Petitjean et al., 2004; Nishiuchi et al., 2017; Yu et al., 2022).

#### 2.2.3 Brucellar spondylitis

Brucellosis is a zoonotic disease caused by Brucella and is one of the most common zoonoses. It can cause multisystem disorders, and spinal involvement may complicate the disease but typically does not lead to death. The ultimate primary cause of death is cardiovascular involvement, with over 80% of fatal cases being associated with Brucella endocarditis (Jin et al., 2023; Spernovasilis et al., 2024). It is a globally prevalent disease, more commonly seen in India, the Mediterranean region, and Mexico, with an incidence ranging from approximately 0.025 to 200 cases per 100,000 population (Guenifi et al., 2025). Brucellosis can not only cause multisystem disorders but also present with a variety of clinical manifestations. It may appear as acute, chronic, or even asymptomatic infection. Common symptoms include fever, fatigue, headache, abdominal pain, malaise, hepatomegaly, etc. Except for fever, which is more prominent in the acute phase, other symptoms are more frequently seen in the subacute and chronic phases (Alp and Doganay, 2008; Spernovasilis et al., 2024). When Brucella involves the spine, it usually presents as either localized or diffuse disease. In localized involvement, osteomyelitis is confined to the anterior endplate at the intervertebral disc–vertebral junction, whereas in diffuse disease, the entire vertebral endplate or the entire vertebral body may be affected (Esmaeilnejad-Ganji and Esmaeilnejad-Ganji, 2019).

#### 2.2.4 Fungal and parasitic spondylitis

Fungal and parasitic infections are uncommon and usually occur in immunocompromised individuals. Common fungal infections include candidiasis and aspergillosis, while parasitic infections are often caused by protozoa and helminths (Skaf et al., 2010; Megaloikonomos et al., 2017; Gonzalez et al., 2023). Symptoms of fungal and parasitic infections are often atypical, with common manifestations including back pain, fever, and fatigue (Zou et al., 2025).

### 2.3 Iatrogenic infection

Iatrogenic infection is caused by direct contact between external pathogens and the spine, usually resulting from surgery or other medical procedures such as puncture (Bose, 2005). Surgery can directly introduce pathogens into the spine, leading to the occurrence and spread of infection (Grossi et al., 2020; Masuda et al., 2023). In addition, it has been reported that invasive conservative treatments such as acupuncture can also cause spinal infections, as these procedures also carry the risk of introducing pathogens into the spine and resulting in infection (Yamashita et al., 1999; Bang and Lim, 2006; Yao et al., 2016). Postoperative infection associated with orthopedic implants is also considered a type of iatrogenic infection. As orthopedic implants are widely used in the treatment of various orthopedic disorders, the spine is no exception. The infection rate following spinal internal fixation reaches 20%, making it the most common cause of unplanned revision surgery within 30 days after primary spinal surgery (Kasliwal et al., 2013; Parchi et al., 2015; Baranowska et al., 2016). Biofilm formation is one of the causes of implant-associated postoperative infections. A biofilm is a structured aggregate of extracellular polysaccharide matrix produced by bacteria themselves, which can adhere to the surface of implants. Implant devices are highly susceptible to bacterial colonization, and even a small number of bacteria can cause infection (Costerton et al., 1999; Donlan, 2001; Verstraeten et al., 2008). Spinal postoperative infection (PSI) can also occur after spinal surgery without implants. The Musculoskeletal Infection Society (MSIS) and the European Bone and Joint Infection Society (EBJIS) have proposed a new definition of PSI that includes six clinical areas: wound characteristics, microbiology, imaging, inflammatory biomarkers, intraoperative findings and histology. Infection can be defined as meeting the independent criterion or three or more major supportive criteria, while suspected infection can be defined as having two major supportive criteria or one major supportive criterion plus two or more minor supportive criteria. The independent criterion is the isolation of phenotypically identical pathogens from two or more deep surgical site specimens by culture. The major criteria include: 1. presence of an inflammatory wound, 2. identification of a pathogen from one deep surgical site specimen, 3. positive blood culture in the absence of other infection sources, 4. intraoperative detection of pus or abscess, 5. imaging evidence of epidural abscess, discitis or vertebral osteomyelitis. The minor criteria include: 1. imaging showing deep fluid accumulation or implant loosening, 2. elevated CRP and/or ESR without other causes, 3. intraoperative findings of pseudarthrosis, implant loosening or breakage, or metallic staining, 4. presence of acute or chronic inflammation (Shaw et al., 2025). According to the time of onset, infections can be classified into early infection, delayed infection and late infection. Early infection refers to infection occurring within 6 weeks after surgery, usually caused by highly virulent bacteria such as *Staphylococcus aureus*, with clinical manifestations of acute infection including acute pain, fever, wound exudation, etc. Delayed infection occurs more than 6 weeks postoperatively, and late infection occurs more than 1 year after surgery. Both are usually caused by low-virulence organisms such as Cutibacterium acnes, and typically present with chronic poor wound healing, fistula formation, implant loosening, and other symptoms (Jagersberg et al., 2020; Palmowski et al., 2020; Prinz and Vajkoczy, 2020).

## 3 Diagnosis

### 3.1 Laboratory examinations

Erythrocyte sedimentation rate (ESR), C-reactive protein (CRP) and white blood cell count (WBC) are the most commonly measured laboratory indicators when spinal infection is suspected. Compared with CRP, ESR and WBC have lower sensitivity and specificity, so CRP is usually the most concerning indicator for clinicians. More than 90% of patients with spinal discitis have elevated CRP levels. In addition, CRP is regarded as the most specific marker for evaluating treatment response, as its level can quickly return to normal after successful treatment (Meyer et al., 1995; Gouliouris et al., 2010; Landi et al., 2019). In addition, studies have shown that a composite wasting phenotype consisting of blood glucose, body temperature, and high-density lipoprotein helps distinguish pyogenic spinal infection from tuberculous infection, which may become a research focus in the future (Wu et al., 2025).

### 3.2 Imaging examinations

Magnetic resonance imaging (MRI) is regarded as the gold standard for the imaging diagnosis of vertebral osteomyelitis. It can clearly demonstrate the extent and severity of infection. In particular, contrast-enhanced MRI can well visualize the scope of soft tissue involvement and abscess formation, and also assess the invasion and compression of surrounding structures (Pingel, 2021; Dhodapkar et al., 2023; Yu et al., 2025). Computed tomography (CT) is often used as an alternative examination for patients with contraindications to MRI, but it also has its own advantages. For example, it can clearly show the degree of vertebral body destruction, more accurately assess bone abnormalities such as endplate and vertebral body erosion, as well as overall bone quality. In addition, CT plays a crucial role in surgical planning (Herren et al., 2017; Pingel, 2021; Dhodapkar et al., 2023). Although X-ray has low specificity, it should be performed for all baseline evaluations (Lener et al., 2018). With continuous advances in nuclear medicine, it has gradually played a role in the diagnosis of spinal infections, especially ^18^F-fluorodeoxyglucose positron emission tomography/computed tomography (^18^F-FDG PET/CT). Numerous studies have demonstrated its increasing importance in diagnosing spinal infections: a negative result can largely rule out the diagnosis of spinal infection, while a positive result can localize active lesions and guide subsequent procedures such as puncture. It can also effectively distinguish spinal infection from Modic changes. Unlike MRI, ^18^F-FDG PET/CT is not affected by metallic implants in the body, so its value is further enhanced in patients with metallic instrumentation. In addition, it can differentiate pyogenic spondylitis from tuberculous spondylitis based on differences in the maximum standardized uptake value (SUVmax), which is generally higher in pyogenic spondylitis (Gemmel et al., 2006; Georgakopoulos et al., 2015; Chen et al., 2023).

### 3.3 Molecular diagnostic techniques

Nucleic Acid Amplification Tests (NAATs) have made it possible to detect trace amounts of bacterial DNA. However, early nucleic acid amplification assays only provided limited information regarding the activity and physiological status of microorganisms detected in samples, and could not distinguish between nucleic acids from viable cells and those from inactivated cells. To address this issue, two novel polymerase chain reaction (PCR)-based strategies have been developed: one is viability PCR, and the other is molecular viability testing (Nocker et al., 2006; Cangelosi et al., 2010; Weigel et al., 2013; Do et al., 2014). Another novel nucleic acid amplification assay based on multiplex PCR technology improves detection sensitivity and enables multiplex real-time PCR testing of vertebral tissue samples. It has been reported to confirm the diagnosis of spinal brucellosis in 90.9% of cases and spinal tuberculosis in 83.3% of cases (Colmenero et al., 2013). Metagenomic next-generation sequencing (mNGS) is an emerging technology with great potential. It has been reported to enable unbiased sampling, allow rapid and broad identification of known pathogens, and even discover new microorganisms (Gu et al., 2019; Miller and Chiu, 2021). Furthermore, the testing time required for mNGS is significantly reduced, and results can be obtained in just 1–2 days (Ramchandar et al., 2021). In a study involving 46 patients, the positive detection rate of mNGS (61.2%) was much higher than that of conventional microbial culture (30.8%). In addition, the required testing time was shorter: conventional microbial culture took (3.09 ± 1.16) days, while mNGS only required (1.54 ± 0.75) days (Chen et al., 2024).

### 3.4 Blood culture and puncture biopsy

Once spinal infection is suspected, blood cultures should be collected before empirical antibiotic therapy, preferably during the peak of fever. Studies indicate that approximately one-third of patients have positive blood cultures, with a diagnostic accuracy of up to 85% for pathogenic bacteria (Patzakis et al., 1991; Nussbaum et al., 1992; Colmenero et al., 1997; Lillie et al., 2008). Some studies also suggest that the positive rate of blood culture generally ranges from 30 to 89%. The wide variation may be related to prior antimicrobial use, duration of bacteremia, regional epidemiology, and other factors. Furthermore, experts recommend that if antibiotics are being administered, they should be discontinued for 1 to 2 weeks before blood culture is performed (Berbari et al., 2015; Nickerson and Sinha, 2016; Dayer et al., 2023). At least two pairs of blood cultures, including aerobic and anaerobic ones, should be obtained. When patients have high-risk factors for Brucella, *Mycobacterium tuberculosis*, or fungal infections, appropriate specific blood cultures should be performed accordingly (Zou et al., 2025). Puncture biopsy is usually performed when blood cultures are negative, as biopsy rarely provides new information when blood cultures are positive (Bae et al., 2018; Kasalak et al., 2021). Recent studies have shown that in patients with suspected spinal infection, the positive rate of CT-guided biopsy ranges from 30 to 35%. Moreover, the microbial detection rate is higher when the punctured tissue is soft tissue, such as intervertebral discs or epidural abscesses (Kim et al., 2015; Dayer et al., 2023). Similar to blood cultures, antimicrobial therapy should be suspended prior to the procedure. Studies have shown that if antibiotics are administered within 48 h before biopsy, the diagnostic positive rate drops from 60 to 23% (de Lucas et al., 2009).

## 4 Therapy

The treatment of spinal infection can generally be divided into conservative treatment and surgical treatment. Conservative treatment is usually the first choice for spinal infection, especially for patients with normal or mildly impaired neurological function, for whom initial conservative treatment is entirely reasonable (Sendi et al., 2008). Antibiotic therapy is a crucial part of conservative treatment. Antibiotic regimens for spinal infection vary considerably depending on the causative pathogen. For pyogenic infections, empirical broad-spectrum antibiotic therapy is generally recommended before the results of drug susceptibility tests are available, to cover the most common Gram-positive and Gram-negative bacteria, such as vancomycin and piperacillin-tazobactam. Once the pathogen is identified, the antibiotic regimen can be adjusted accordingly (Henry et al., 2025). The duration of treatment is usually 6–12 weeks. Antibiotics may be administered intravenously for the initial 2–4 weeks, followed by oral therapy. Studies have shown that a short course (6–8 weeks) of antibiotic treatment is usually sufficient for patients at low risk of recurrence, while those at high risk require an extended course (>8 weeks). For infections caused by *Staphylococcus aureus*, a longer treatment course remains the preferred option due to the high treatment failure rate (Lener et al., 2018; Zou et al., 2025). Some studies also suggest that a 6-week antibiotic course is equally effective as a 12-week course for pyogenic spondylitis (Bernard et al., 2015). Tuberculous spondylitis requires an anti-tuberculosis regimen. An intensive phase of isoniazid, rifampicin, ethambutol and pyrazinamide is administered for the initial 2 months, followed by adjustment of the consolidation phase medications according to drug susceptibility results. In the absence of susceptibility data, isoniazid, ethambutol and rifampicin are continued for 12 months. If results are available, treatment with two agents is sufficient. For strains susceptible to isoniazid and rifampicin, therapy is continued for 12 months; for those susceptible to other agents, treatment is extended to 18–24 months (Quiñones-Hinojosa et al., 2004). The greatest difference between the treatment of nontuberculous mycobacterial infection and tuberculous mycobacterial infection is that tuberculous mycobacterial infection can usually be successfully cured with antimicrobial therapy alone, whereas successful treatment of nontuberculous mycobacterial infection often relies on aggressive surgical debridement. In addition, since *in vitro* drug resistance of nontuberculous mycobacteria does not equate to *in vivo* drug resistance, drug susceptibility test results must be interpreted by mycobacterial specialists. Amikacin resistance generally indicates a poor prognosis (Wessell et al., 2024). For spinal infection caused by Brucella, literature has shown that doxycycline combined with streptomycin is an effective treatment regimen, but in clinical practice, doxycycline combined with gentamicin is the more recommended option. Fungal infections usually require long-term treatment with azoles or amphotericin B (Deng et al., 2019; Guenifi et al., 2025; Zou et al., 2025). Spinal infection caused by parasites is relatively rare. Antiparasitic agents such as albendazole, mebendazole, or praziquantel are recommended for treatment. Hydatid disease differs from other parasitic infections in its management, with surgical treatment being the first choice (Majmundar et al., 2019; Sioutis et al., 2021). However, not all patients improve with conservative treatment. We recommend surgical treatment for patients presenting with the following conditions: spinal instability or deformity, neurological deficits, failure of conservative treatment, intraspinal infection, and severe systemic infection such as sepsis. The basic principles of surgical treatment are: maintaining spinal stability, debriding infected tissues, identifying pathogenic bacteria, and ensuring sufficient blood flow at the lesion site to promote tissue healing (Muller et al., 2004; Gonzalez et al., 2023; Henry et al., 2025; Yu et al., 2025; Zou et al., 2025). For surgical strategies in spinal infection, one-stage radical debridement and reconstruction is a widely accepted principle. Surgical approaches can be classified into posterior-only approach, anterior-only approach, and combined anterior–posterior approach. The selection of surgical approach depends on various factors, including the location of infection, characteristics of the pathogenic bacteria, and the extent of tissue involvement, among others. Anterior plating is typically indicated for cervical spine cases, while additional posterior stabilization is required for multilevel involvement. In thoracolumbar spine patients, one-stage posterior decompression, debridement combined with internal fixation often achieves satisfactory outcomes. If adequate results cannot be obtained via a posterior-only approach, a combined anterior–posterior procedure may be performed (Muller et al., 2004; Henry et al., 2025; Yu et al., 2025). Secondly, even for the same disease such as pyogenic spondylitis, different treatment options may be selected based on varying severity. In such cases, proper classification provides guidance for treatment. Pola classified pyogenic spinal infections according to three major criteria (bone destruction or segmental instability, epidural abscess, and neurological impairment) and two minor criteria (paravertebral soft tissue involvement and intramuscular abscess). Sonawane classified thoracolumbar tuberculosis based on four scoring criteria: pain intensity (quantified by the VAS score), kyphotic angle, degree of vertebral destruction, and neurological status. Pluemer also classified spinal infections based on four variables: neurological deficit, site of infection, imaging findings on CT and MRI, and degree of pain and presence of comorbidities. Clinicians can then select the optimal treatment plan according to the different categories (Pola et al., 2017; Pluemer et al., 2023; Sonawane et al., 2024). Minimally invasive spine surgery (MISS) is also being gradually promoted. MISS offers advantages including less surgical trauma, reduced infection spread, less intraoperative bleeding, faster postoperative fusion, and fewer postoperative complications. For example, the thoracoscopic approach has been proven effective in the treatment of thoracic spine infections. For patients with single-level thoracolumbar pyogenic spondylodiscitis and high functional demands, percutaneous screw-rod internal fixation can be an alternative to brace immobilization or a standard procedure following anterior debridement. The oblique lumbar corridor has also been successfully used to treat single-level infections of the upper and middle lumbar spine, and the lateral transpsoas approach has achieved similar success (Turunc et al., 2007; Deininger et al., 2009; Duarte and Vaccaro, 2013; Tuchman et al., 2014; Pola et al., 2017; Campbell et al., 2023). With regard to postoperative infection, the retention or removal of implants is a critical concern. In early infection where biofilms remain immature, thorough early debridement can effectively eliminate pathogens, allowing implants to be retained. In delayed or late infection, mature biofilms are generally present and cannot be completely eradicated from the implant surface, thereby necessitating implant removal and replacement (Kalfas et al., 2019; Prinz and Vajkoczy, 2020). Nevertheless, the decision to retain or remove implants is influenced by multiple additional factors, including concomitant sepsis, long-segment fusion involving more than three levels, preoperative hypohemoglobinemia, MRSA infection, obvious intervertebral abscess on MRI, and delayed administration of sensitive antibiotics (Núñez-Pereira et al., 2013; Tominaga et al., 2016; Kanayama et al., 2017).

## 5 Summary

With the aging of the population and other contributing factors, the incidence of spinal infection has gradually increased. Early diagnosis is crucial for spinal infection. However, due to its non-specific clinical manifestations, delayed diagnosis is not uncommon. The treatment course is relatively long, and multidisciplinary cooperation is required to ensure appropriate management. Active surgical intervention should be performed once surgical indications are confirmed.
